# Supplementary material for: Modeling the electrical resistivity of polymer composites with segregated structures
Source: Nat Commun. 2019 Jun 10;10:2537. doi: 10.1038/s41467-019-10514-4 (PMC6557821; doi:10.1038/s41467-019-10514-4)
Supplement: Supplementary file 1 — Supplementary information [file 41467_2019_10514_MOESM1_ESM.pdf]

Supplementary information

Modeling the electrical resistivity of polymer composites with segregated structures

Park et al.

Supplementary Figure 1 How to generate a single random nanowire

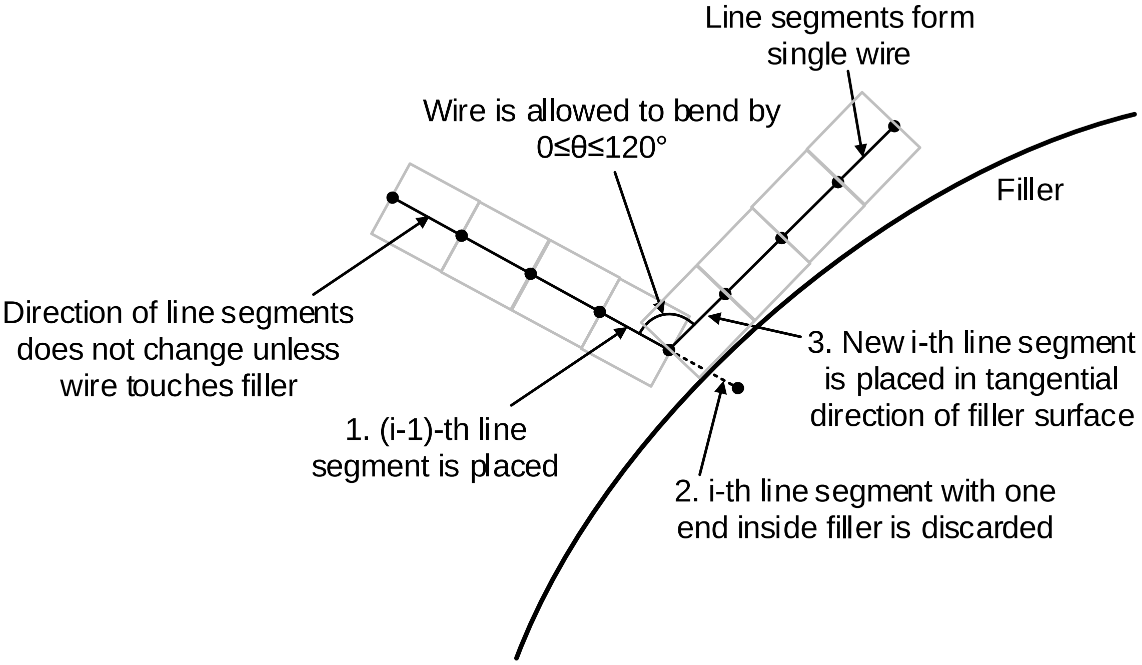

Supplementary Figure 2 How to determine whether two nanowires have a contact

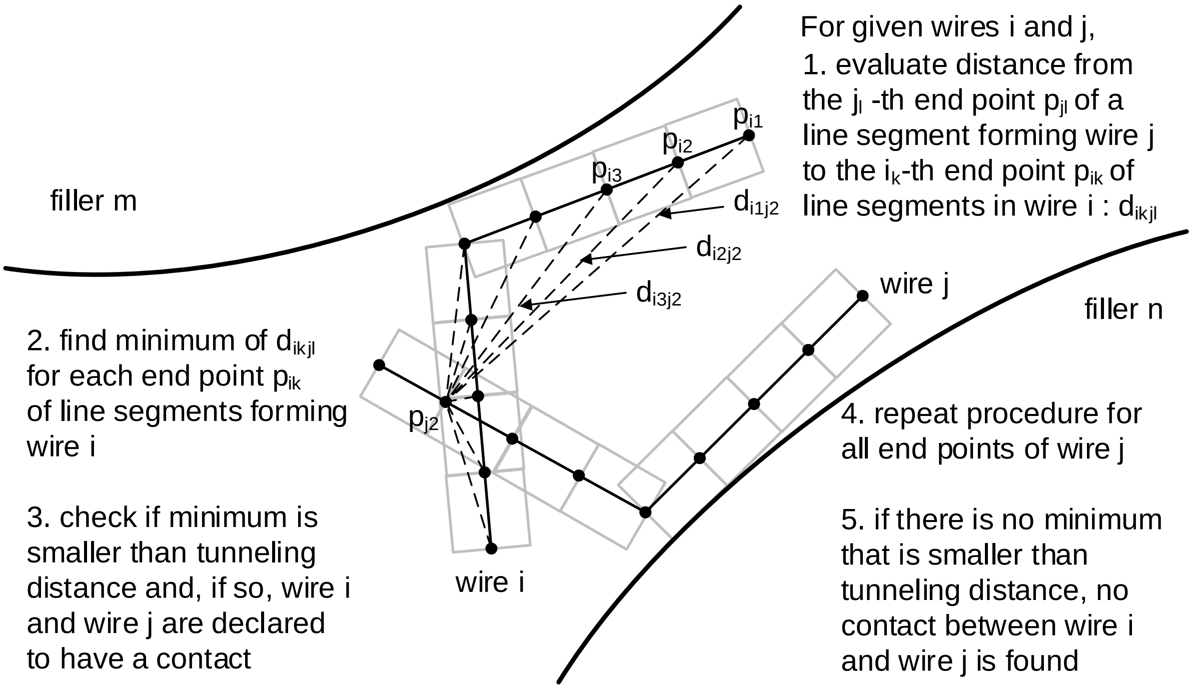

Supplementary Figure 3 Distributions of CNT length before/after three-roll milling

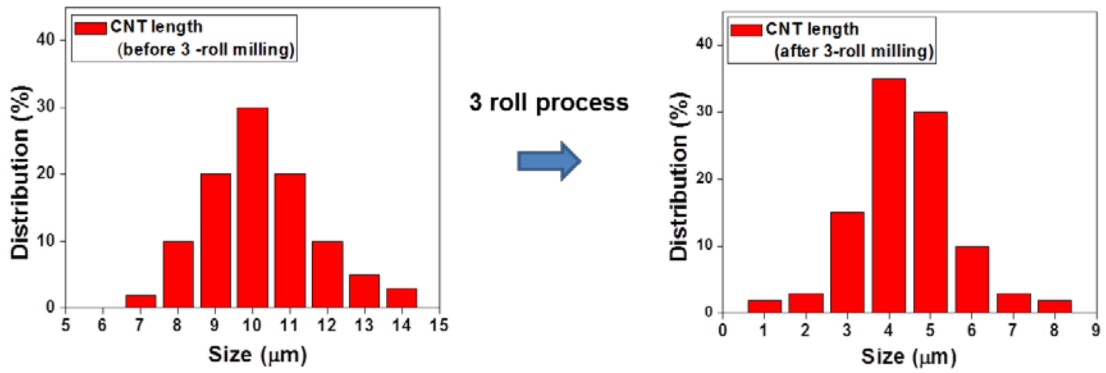

Supplementary Figure 4 SEM images of CNT before/after three-roll milling

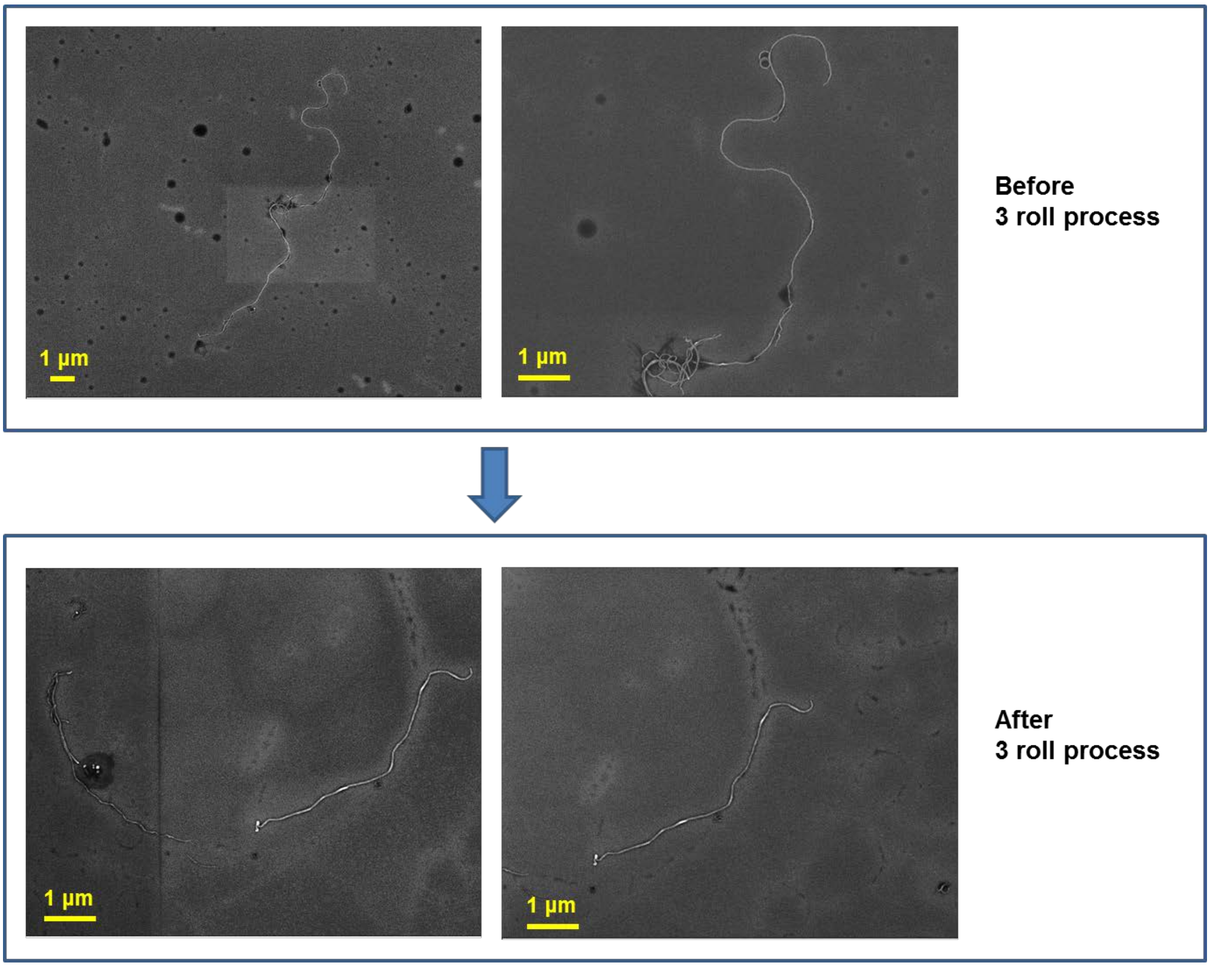

Supplementary Figure 5 Distributions of micro-silica particle size before/after three-roll milling

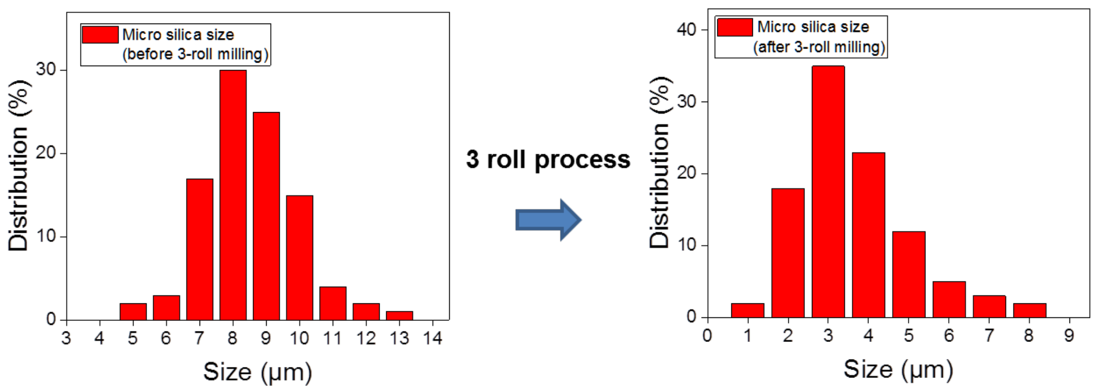

Supplementary Figure 6 SEM images of micro-silica particles before/after three-roll milling

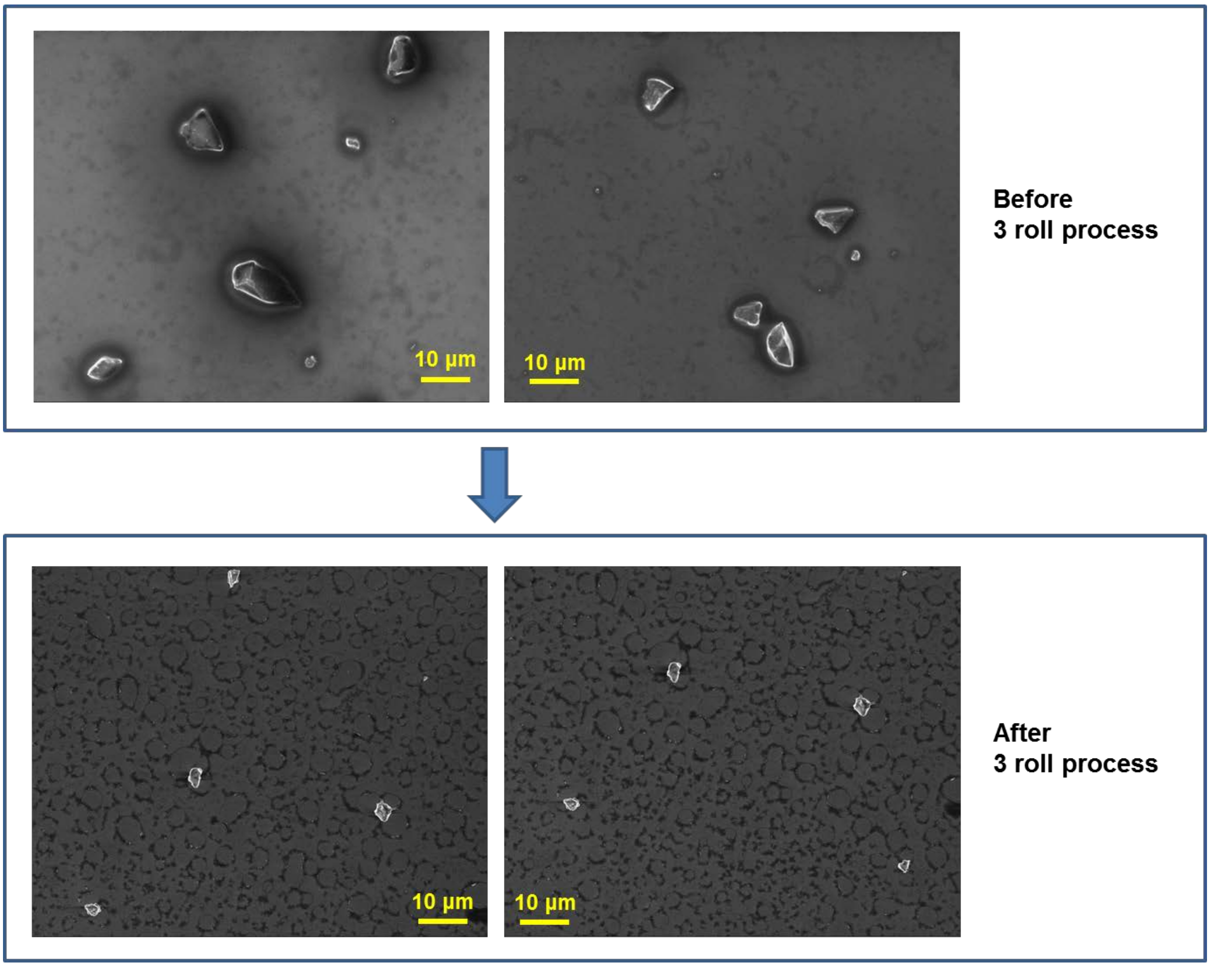

Supplementary Figure 7 (a) Raman spectra of the CNT/Silica/PDMS composites at a wavelength of 514.5 nm to determine changes in the D band (defect and disorder) and the G band (carbon structure). CNT 1 wt% only, CNT 1wt% with micro silica (40 wt%) and CNT 1 wt% with nano silica (30 wt%) are shown. (b) Raman spectra with full range (0 to 3000 cm^{-1})

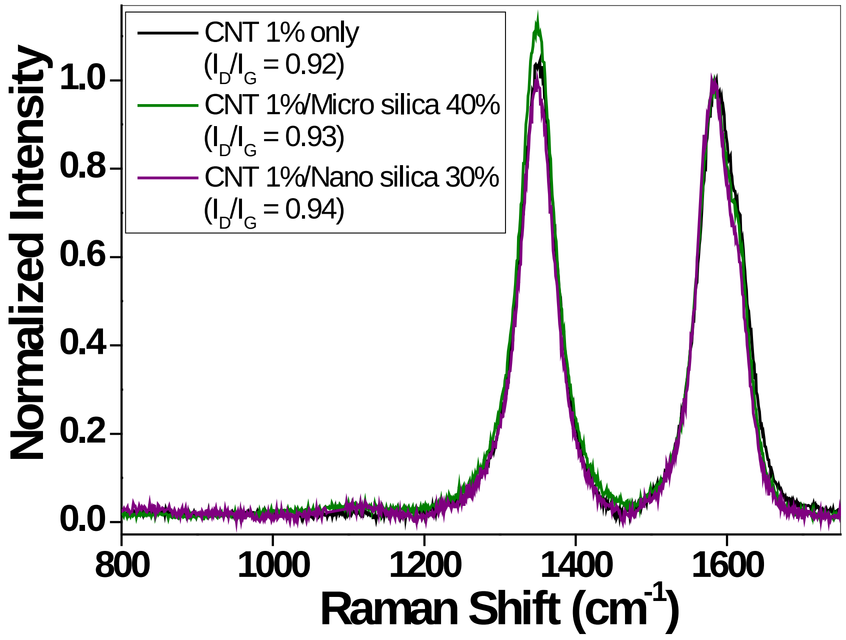

(a)

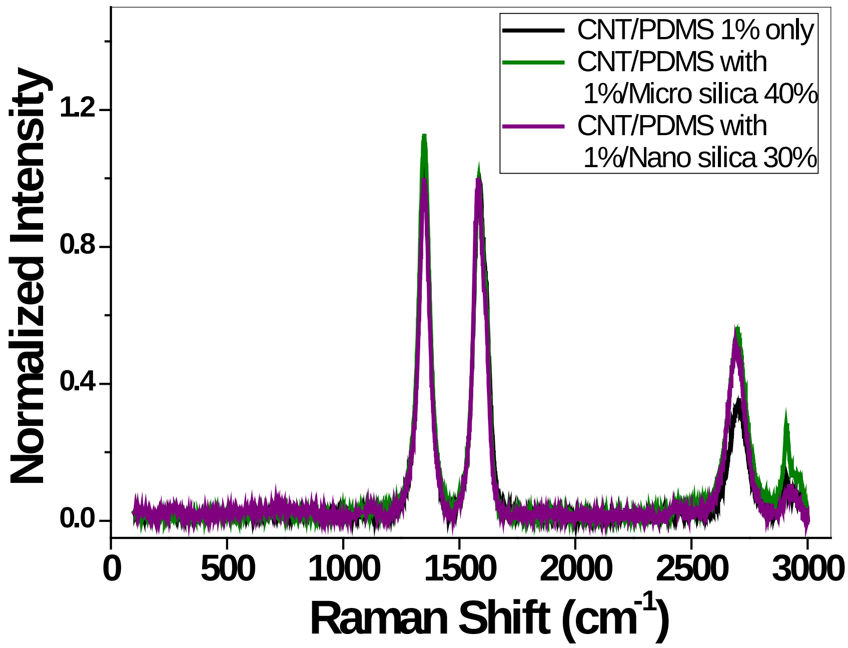

(b)

Supplementary Figure 8 The DC electrical conductivity (σ_{DC}) of the CNT composites follows a power law characteristic of percolation-like behavior ($\sigma_{DC} \sim \sigma_0(p-p_c)^{\beta}$). The inset shows a log–log plot of σ versus $(p - p_c)/p_c$. From the inset figures, critical exponent (β) was evaluated ((a) composite with nano-silica is 2.7, (b) composite without silica is 1.6 and (c) composite with micro-silica is 1.2).

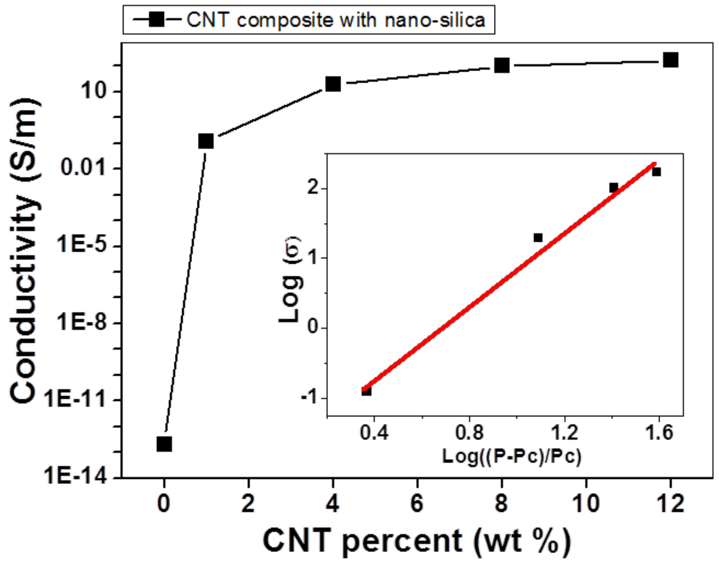

(a)

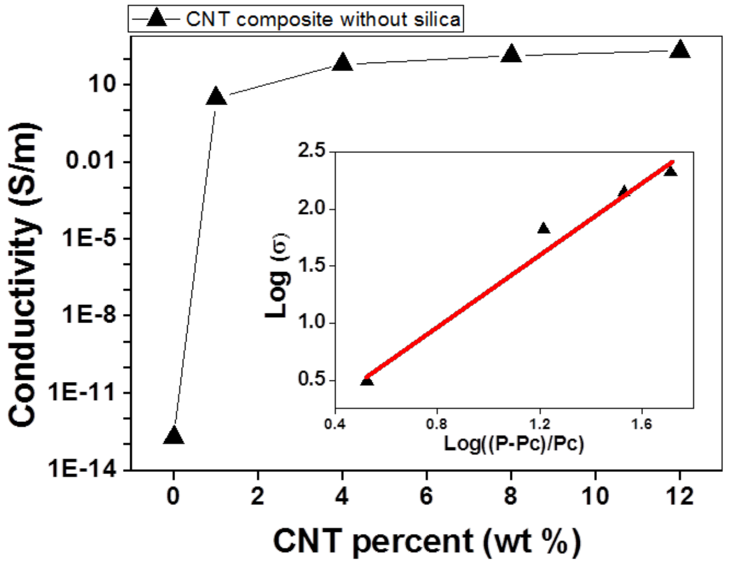

(b)

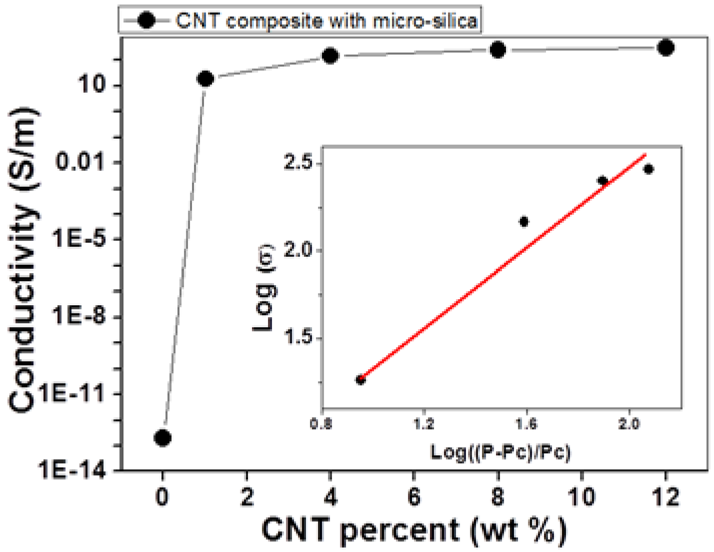

CNT composite with micro-silica
Conductivity (S/m)
10
0.01
1E-5
1E-8
1E-11
1E-14
0
2
4
6
8
10
12
CNT percent (wt %)
Log (σ)
2.5
2.0
1.5
0.8
1.2
1.6
2.0
Log((P-Pc)/Pc)

(c)

Supplementary Figure 9 Simulation images in case of CNT networks with nano-size sphere (filler diameter = 250 nm) with 25μm×25μm domain size

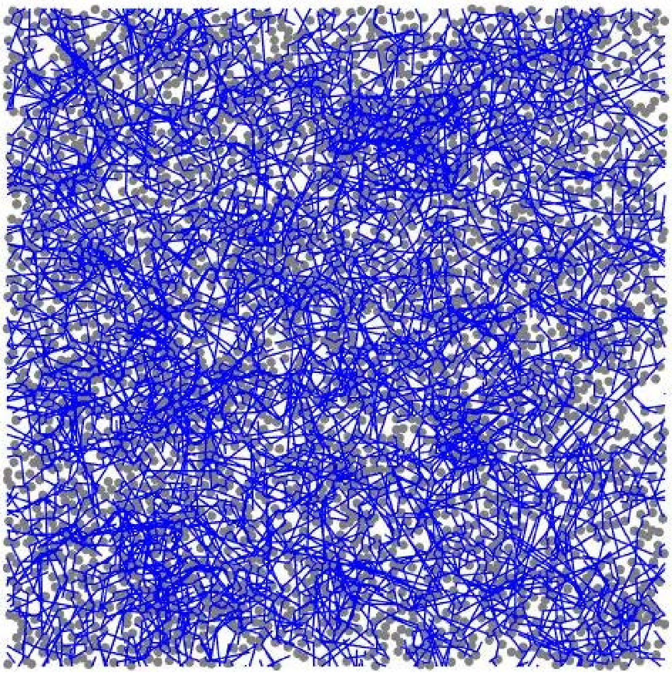

Supplementary Note 1 Characterization of tunneling mechanism

In the 3-D Monte-Carlo simulation, the tunneling resistivity (ρ_c) between CNTs is estimated by [S1]

$$\rho_c = \frac{h^2}{e^2\sqrt{2m\lambda}}\exp\left(\frac{4\pi d}{h}\sqrt{2m\lambda}\right) \quad (1)$$

where h is the Planck's constant, e is the electric charge of electron, m is electron mass, d is the tunneling distance between CNTs, and λ is the tunneling barrier height which set to 0.5 eV. The maximum value of the tunneling distance d is assumed to be 2 nm implying that two CNTs closer than 2 nm are assumed to be electrically connected. [S2-S3] The contribution of CNT resistivity is considered to be negligible as compared to the contact resistance that it is not considered in this simulation. [S4]

Supplementary Figure 10 Simulation images of CNT networks (a) without silica, (b) with micro-size sphere (filler diameter = 4 μm), and (c) with nano-size sphere (filler diameter = 20 nm). (d) Magnified image of the area marked with red square in (c). (a) - (c) are same domain size (25μm×25μm), and domain size of (d) is 1.75μm×1.75μm.

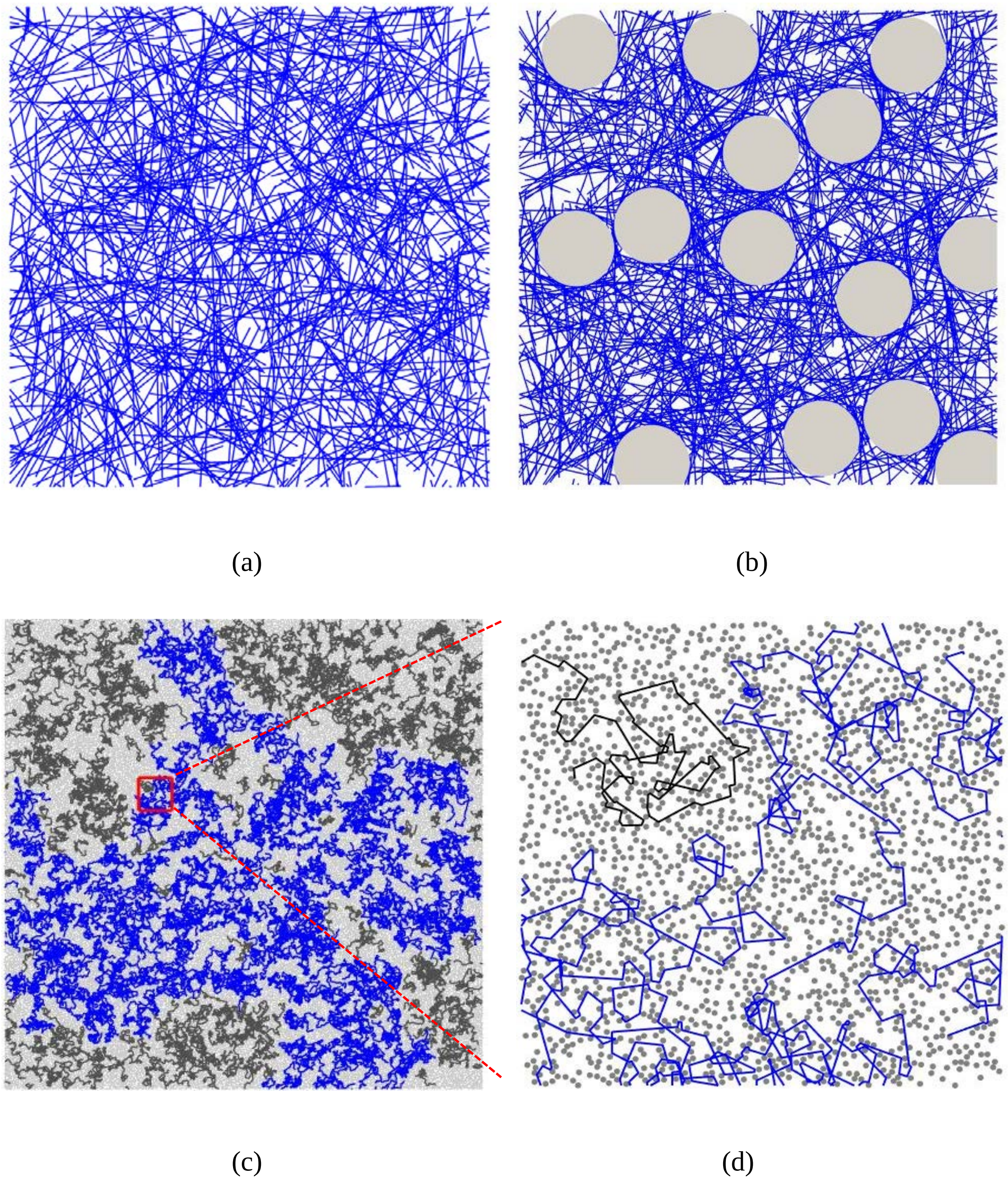

Supplementary Figure 10 (a) shows a random network of CNTs with a diameter of 10 nm and a length of 5 μm leading to the sheet resistance of 340 Ω sq^{-1}, which is comparable to the resistance value with the experimental results. **Supplementary Figure 10 (b)** and **(c)** are representative random instances of the CNT/silica composites generated with the same loading amount of CNTs in **Supplementary Figure 10 (a)** along with the micro-sized (a diameter of 4 μm) and the nano-sized (a diameter of 20 nm) particulate fillers, respectively, with an area coverage of 20 %. As compared to the CNT network without silica fillers in shown in **Supple mentary Figure 10 (a)**, the CNT network formed in the CNT/micro-sized silica composite provides several dense CNT clusters owing to the excluded area arising from the micro-sized silica fillers, which is consistent with the excluded volume theory discussed in previous studies. The topological change of the network leads to the enhancement in the probability of CNTs being interconnected as the number of junctions connected to other CNTs on a single CNT increases after incorporating the micro-sized silica. The increase in the number of interconnecting junctions in the percolating network is an explicit evidence of the resistivity reduction of the network.

On the other hand, in the composite of CNT and nano-sized silica with a diameter of 20 nm, a number of curly CNTs are entangled in the network as shown in **Supplementary Figure 10 (c)**. Clearly, the densely-dispersed nano-sized particulate fillers cause severe bending of CNTs as presented in the **Supplementary Figure 10 (d)**, the enlarged figure of the area marked by the red rectangle in **Supplementary Figure 10 (c)**. The modification of the network topology leads to the probability reduction of CNTs forming a conducting network as the number of interconnecting junctions on a single CNT decreases. Moreover, several interrupted or isolated paths are discovered (CNTs drawn in gray color in **Supplementary Figure 10 (c)**), which do not participate in electrical conduction. This result corresponds to the resistivity increase of the CNT/nano-sized silica composite measured in experiment.

Supplementary Figure 11 (a) Simulated normalized resistance (R/R_0) as a function of silica wt% for different silica size. The values are matched to experimental results with CNT contents fixed at 1 wt% (b) Resistance difference (ΔR) as a function of CNT wt% for different silica size with fixed silica content (36 vol%). Horizontal line indicates CNT composite without silica particle.

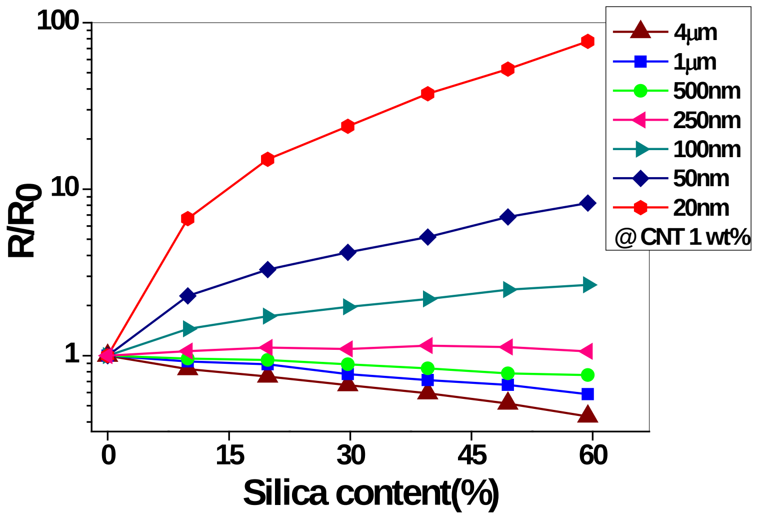

(a)

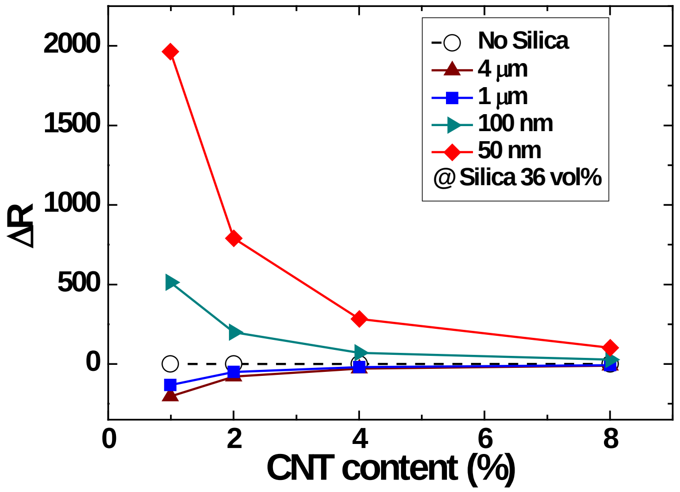

(b)

The resistance change of the CNT network caused by the modification of the network topology is calculated as shown in **Supplementary Figure 11 (a)**. According to the calculation results, for the fixed amount of particulate fillers, the composites with the incorporating fillers with larger diameters have the advantage of obtaining a lower sheet resistance. In addition, for the case of particulate fillers with diameter larger than 250 nm, the resistance of CNT networks decreases as the amount of incorporating silica increases. By contrast, in the case of particulate fillers with diameter smaller than 250 nm, the resistance of the CNT network increases as more silica particles are incorporated in the composite. A random instance of CNT/silica composite with silica particles with a diameter of 250 nm is presented in **Supplementary Figure 9**. The CNT network topology of the composite is analogous to the CNT network without silica particles shown in **Supplementary Figure 10**, indicating that the silica particles with a diameter of 250 nm do not affect the network topology. Thus, the resistance of the CNT network remains unchanged with respect to the addition of the silica particles, which is in accordance with the results in **Supplementary Figure 11 (a)**. Furthermore, as can be seen from the resistance results calculated under the fixed particulate filler content shown in **Supplementary Figure 11 (b)**, the simulation results also present pronounced effects of particulate fillers on the resistivity of the CNT/silica composites under lower CNT loading, which corresponds to experimental results.

Supplementary References

[S1] Hu, N., Karube, Y., Yan, C., Masuda, Z., & Fukunaga, H. Tunneling effect in a polymer/carbon nanotube nanocomposite strain sensor. *Acta Mater*. **56**, 2929-2936 (2008).

[S2] Li, C., Thostenson, E. T., & Chou, T. -W. Dominant role of tunneling resistance in the electrical conductivity of carbon nanotube–based composites. *Appl. Phys. Lett.* **91**, 223114 (2007).

[S3] Lu, W., Chou, T. -W., & Thostenson, E. T. A three-dimensional model of electrical percolation thresholds in carbon nanotube-based composites. *Appl. Phys. Lett.* **96**, 223106 (2010).

[S4] Yu, Y., Song, G. & Sun, L. Determinant role of tunneling resistance in electrical conductivity of polymer composites reinforced by well dispersed carbon nanotubes, *J. Appl. Phys.* **108**, 084319 (2010).
